# Evaluation of Stool Short Chain Fatty Acids Profiles in the First Year of Life With Childhood Atopy-Related Outcomes

**DOI:** 10.3389/falgy.2022.873168

**Published:** 2022-04-06

**Authors:** Hsin Yue Cheng, James Chun Yip Chan, Gaik Chin Yap, Chiung-Hui Huang, Dorinda Yan Qin Kioh, Elizabeth Huiwen Tham, Evelyn Xiu Ling Loo, Lynette P. C. Shek, Neerja Karnani, Anne Goh, Hugo P. S. Van Bever, Oon Hoe Teoh, Yiong Huak Chan, Christophe Lay, Jan Knol, Fabian Yap, Kok Hian Tan, Yap-Seng Chong, Keith M. Godfrey, Eric Chun Yong Chan, Bee Wah Lee, Le Duc Huy Ta

**Affiliations:** ^1^Department of Paediatrics, Yong Loo Lin School of Medicine, National University of Singapore, Singapore, Singapore; ^2^Singapore Institute of Food and Biotechnology Innovation, A^*^STAR, Singapore, Singapore; ^3^Skin Research Institute of Singapore, A*STAR, Singapore, Singapore; ^4^Department of Pharmacy, Faculty of Science, National University of Singapore, Singapore, Singapore; ^5^Khoo Teck Puat-National University Children's Medical Institute, National University Health System, Singapore, Singapore; ^6^Singapore Institute for Clinical Sciences (SICS), Agency for Science, Technology and Research (A*STAR), Singapore, Singapore; ^7^Department of Paediatrics, KK Women's and Children's Hospital, Singapore, Singapore; ^8^Biostatistics Unit, Yong Loo Lin School of Medicine, National University of Singapore, Singapore, Singapore; ^9^Danone Nutricia Research, Singapore, Singapore; ^10^Danone Nutricia Research, Utrecht, Netherlands; ^11^Laboratory of Microbiology, Wageningen University, Wageningen, Netherlands; ^12^Department of Obstetrics and Gynaecology, National University of Singapore, Singapore, Singapore; ^13^MRC Lifecourse Epidemiology Centre and NIHR Southampton Biomedical Research Centre, University of Southampton and University Hospital Southampton NHS Foundation Trust, Southampton, United Kingdom

**Keywords:** stool SCFA, eczema, wheezing, allergen sensitization, GUSTO

## Abstract

**Introduction:**

Short chain fatty acids (SCFAs) are the main intestinal intermediate and end products of metabolism of dietary fibers/polyphenols by the gut microbiota. The aim of this study was to evaluate the biological implication of stool SCFA profiles determined in the first year of life on the clinical presentation of allergic outcomes in childhood.

**Methods:**

From the Growing Up in Singapore Toward healthy Outcomes (GUSTO) cohort, a sub-cohort of 75 participants was recruited. Scheduled questionnaire data was collected for cumulative prevalence of physician-diagnosed eczema, wheezing with the use of nebuliser, and allergen sensitization till the age of 8 years. Stool samples collected at week 3 and months 3, 6 and 12 were quantitated for 9 SCFAs using LC/MS/MS. SCFA data were grouped into lower (below the 25th^)^ and higher (above the 75th percentiles) categories. Generalized Linear Mixed Models was employed to analyse longitudinal association between SCFAs and atopy-related outcomes.

**Results:**

Children with lower stool butyric acid levels (≤25th percentile) over the first 3 time points had higher odds ratio (OR) for wheezing (adjOR = 14.6), eczema (adjOR = 13.2), food sensitization (adjOR = 12.3) and combined outcomes of both wheezing and eczema (adjOR = 22.6) till age 8 years, compared to those with higher levels (≥75 percentile). Additionally, lower longitudinal levels of propionic acid (≤25th percentile) over 4 time points in first year of life was associated with recurrent wheezing (≥2 episodes) till 8 years (adjOR = 7.4) (adj *p* < 0.05).

**Conclusion:**

Our results suggest that relatively low levels of gut SCFAs in early life are associated with increased susceptibility to atopic-related outcomes in childhood.

## Introduction

The prevalence of allergic diseases has increased in the recent decades and is forecasted to continue to increase worldwide (1). Current evidence suggests that a perturbation of gut microbiota influences the development of allergic diseases (2). The human gut microbiota is involved in various metabolic processes that collectively regulate immunity and gut health. Infancy is an important stage for the establishment and maturation of the gut microbiome. Factors such as gestational age, mode of delivery, antibiotics exposure, feeding modes, environmental exposures and host genetics are known to influence this maturation process (2).

Short chain fatty acids (SCFAs) are derived from gut microbial fermentation of dietary fibers and polyphenols. These metabolites play a key role in maintaining gut and immune homeostasis (3, 4). They are absorbed in the colon and exert systemic effects by regulating various physiological processes and acting as signaling molecules, maintaining balance between pro- and anti-inflammatory properties (3). Two main anti-inflammatory signaling mechanisms of SCFAs have been identified: inhibition of histone deacetylases (HDACs), and activation of G-protein-coupled receptors (GPCRs) (4, 5). In addition, SCFAs have also been found to potentiate *de novo* extrathymic Treg cell generation (4). In support, seven prospective birth cohort studies have demonstrated that infants with higher stool SCFAs, particularly butyric acid have less atopic outcomes in childhood (6–12).

This study leveraged data from a sub-cohort from the Growing Up in Singapore Toward Healthy Outcomes (GUSTO) birth cohort to evaluate the biological implication of infant stool SCFA profiles on clinical outcomes of atopic eczema, wheezing and allergen sensitization up to the age of 8 years. To investigate the protective role of SCFAs against the development of atopic disorders, serial infant stool SCFA measurements at 4 time points (3 weeks, 3, 6, and 12 months) were longitudinally analyzed in order to determine their potential influence on atopic outcomes in childhood.

## Methods

### Study Design

The GUSTO cohort is Singapore's largest and most comprehensive birth cohort study which aims to evaluate whether mothers' diet and lifestyle during pregnancy would affect their babies' growth and development after birth. Of the 1,237 Infants born to enrolled mothers, 333 infants were excluded due to dropouts during follow-up. Seventy-five of the remaining 904 infants who were included in this study had allergy-related data from birth up to the 8-year time-point. Additionally, from those seventy-five infants, stool samples collected at 3 weeks, 3, 6 and 12 months were analyzed for different parameters as described previously (13). Ethics approval was obtained from the Domain Specific Review Board of Singapore, National Healthcare Group and the Centralized Institutional Review Board of SingHealth (DSRB D/09/021 and CIRB 2009/280/D). Informed written consent was obtained from all subjects.

Interviewer-administered questionnaires and scheduled follow-up visits were carried out at 3 weeks, 3, 6, 9, 12, 15, 18 months, 2, 3, 4, 5, 6 and 8 years. Data was collected on demographics, family history of atopic disorders, social and lifestyle factors, as well as detailed clinical data on infant allergy-related outcomes.

A physician's diagnosis of eczema in the child was determined by a positive answer to the written question: “Has your child ever been diagnosed with eczema?”. Wheezing was defined as the presence of wheeze symptoms (noisy breathing with a high-pitched, whistling sound heard from the chest, not the mouth) and with the use of nebulizer. Recurrent wheeze was defined as the presence of wheeze symptoms at more than one time point. Children with both eczema and wheezing were defined as subjects who developed both eczema and wheeze at any time point during follow up period.

Allergen sensitization was assessed through skin prick testing (SPT) to aeroallergens (house dust mites *Dermatophagoides pteronyssinus, Dermatophagoides farinae* and *Blomia tropicalis* at 18 months, 3, 5, and 8 years, and cockroaches *Blatella germanica* and *Periplaneta americana* at 8 years only) and to food allergens (egg, peanut and cow's milk) at the 18-month, 3, 5, and 8-year visits. These are the most common aeroallergens and food allergens in sensitized Singaporean children (14). All skin prick extracts were obtained from Greer Laboratories (Lenoir, NC, USA), except for *B. tropicalis*, which was obtained from our in-house laboratory. *B. tropicalis* extract was prepared as previously described (15). A wheal of at least 3 mm was defined as a positive SPT and a child was considered as SPT-positive (allergen-sensitized) if any one or more of the individual tests were positive with a positive reaction to histamine (positive control) and negative reaction to saline (negative control).

Food allergy (milk, egg, soy, wheat, fish, peanut, tree nut and shellfish) was defined as a positive SPT of ≥3 mm to the specified food and a convincing history of an IgE-mediated reaction upon exposure to the food.

### SCFA Quantification

Stool samples were collected at 3 weeks, 3, 6, and 12 months at each subject's home, and ranged from 2 to 20g of stool each time. Stool samples were quantified for 9 SCFAs (acetic, propionic, butyric, isobutyric, valeric, isovaleric, 2-methylbutyric, caproic and 4-methylvaleric acids) by liquid chromatography tandem mass spectrometry (LC/MS/MS). Samples were stored at −20°C upon collection and was then stored at −80°C once transported to the laboratory. The stool was stored between 4 and 6 years prior to analysis. The methodology of SCFA quantification were performed as reported previously (16). The number of stool samples quantified at each time point were 28, 41, 58, and 61 respectively.

### Statistical Analysis

All data analysis was conducted using IBM SPSS Version 25. SCFA data were stratified as below the 25th and above the 75th percentiles for comparison to draw out clearer associations and significance with allergy-related outcomes between two extreme quartiles (11). Linear regression was employed to analyse the significance of the trend of cumulative allergy-related outcomes and SCFA concentration levels over time. Logistic regression was used to analyse the association between SCFA concentration at each time point with the allergy-related outcomes, whereas Generalized Linear Mixed Models (GLMM) was employed to analyse the longitudinal association between stool SCFAs across all time points and the cumulative allergy-related outcomes (Supplementary Tables 1–3). The analysis was adjusted for confounders, namely gender, presence of siblings, mode of delivery, family history of atopic diseases and feeding pattern (exclusively breastfeeding, partially breastfeeding or exclusively formula in the first 6 months of life). All statistical significance tests and confidence intervals (CIs) were 2-sided and set at a *p*-value of < 0.05.

## Results

### Demographic and Clinical Characteristic of Subjects

Our subjects (*n* = 75) were of Chinese (56.0%), Malay (28.0%) and Indian (16.0%) ethnicity. They were 50.7% male, 61.3% had siblings, 33.3% of infants were delivered *via* Cesarean-section, and 46.4% had a family history of allergy. The infant feeding pattern varied from exclusively breastfeeding (4.0%), mixed feeding (84.0%), to never breastfeeding (12.0%). Majority of the infants (96.0%) were born at full term. 32.4% of mothers received intrapartum antibiotics, and 23.0% of infants consumed antibiotics in the first year of life (Supplementary Table 4).

Compared to the remaining larger cohort (*n* = 829) which was not included in this study. The demographic and clinical characteristics, including allergy-related outcomes, between this selected sub-cohort of subjects (*n* = 75) were not significantly different, except for higher post-natal antibiotics use within the first year of life and a higher proportion of exclusively breastfed infants in the larger cohort (*p* < 0.05) (Supplementary Table 4).

### Cumulative Prevalence of Allergy-Related Outcomes

Within the sub-cohort of 75 evaluated in this study, the 8 years cumulative prevalence for eczema was 35/68 (51.5%), 19/62 (30.6%) for wheezing, 9/62 (14.5%) for recurrent wheeze, 45/64 (70.3%) for inhalant sensitization, 17/63 (27.0%) for food sensitization, 45/65 (69.2%) for any sensitization, and 16/46 (34.8%) for combined eczema and wheezing outcomes. The denominators for cumulative prevalence of each allergy-related outcomes varied due to missing data. Missing data for cumulative allergy-related outcomes is defined where subjects had missing data at any time point and answered “no” for the other time points. Clinical food allergy was seen in 9 of the 75 subjects (12.2%) (Supplementary Table 4). In view of the relatively small size of food allergic subjects, further analysis of this group was not performed. There was no significant difference in demographic factors between those with and without allergy-related outcomes except for the paternal history of allergic diseases (*p* < 0.05) (data not shown).

**Figure 1:**
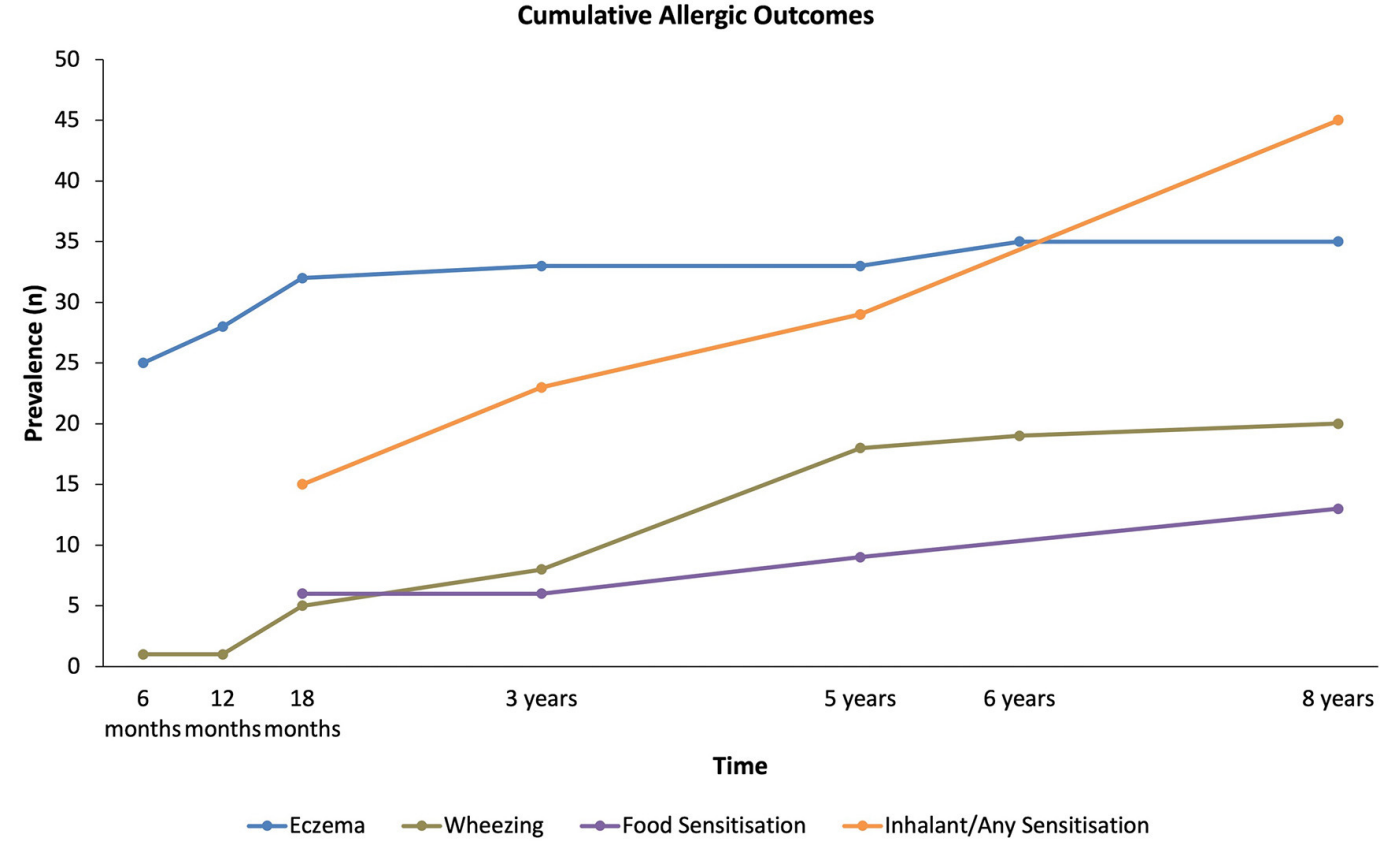
Cumulative prevalence for all allergy-related outcomes up to 8 years of age. The trends for the increase in cumulative prevalence of each allergy-related outcomes over time were all significant (*p* < 0.05).

The cumulative number of subjects who developed eczema, wheezing as well as subjects who were sensitized to food or inhalant allergens with time is shown in Figure 1. The rate of increase of cumulative prevalence of allergy-related outcomes increased significantly with age (*p* < 0.05). Eczema manifested early with prevalence of 33.8% at month 6 and thereafter increased slightly to 51.5% at 8 years of age, whereas wheezing continued to increase steadily from 18 months to 4 years. Food sensitization showed a gradual increase from 3 to 8 years, while inhalant sensitization increased sharply from 18 months to 8 years.

### Stool SCFA Concentration Variation With Time

**Figure 2:**
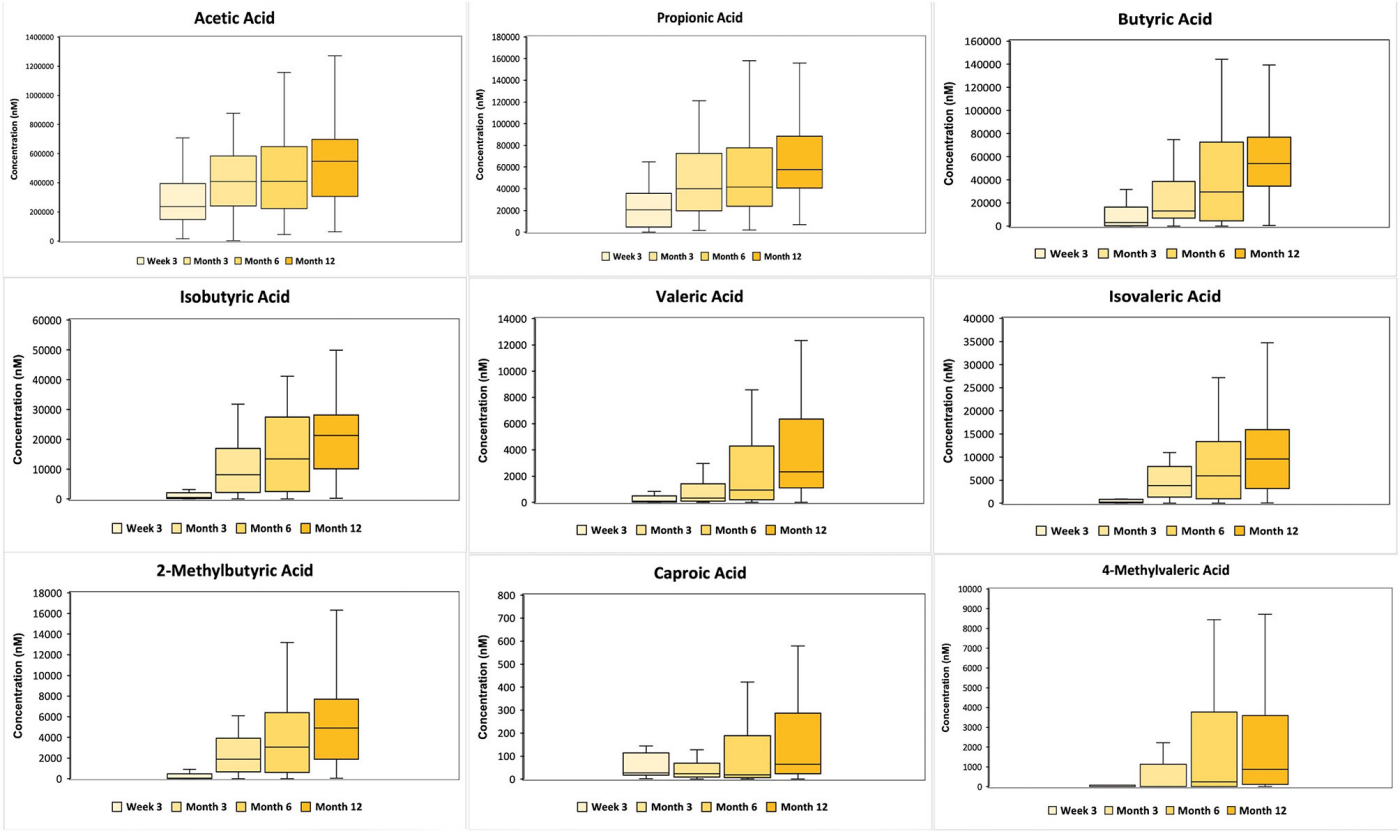
10th to 90th percentile SCFA concentration (in nanomolar) of 6 major SCFAs (acetic, propionic, butyric, isobutyric, isovaleric and caproic acid) and 3 minor SCFAs (valeric, 2-methylbutyric and 4-methylvaleric acid) across 3 weeks, 3, 6 and 12 months, where the middle refers to the median value. The major and minor SCFAs are split according to their concentrations. The concentration of all SCFA concentration, except caproic and 4-methylvaleric acids, increased significantly with time (*p* < 0.05).

Figure 2 shows the SCFA concentrations in stool samples across 4 time points (3 weeks, 3, 6 and 12 months). The concentration of all SCFAs increased significantly with time (*p* < 0.05), except for caproic and 4-methylvaleric acids where the increase over the first year was marginal.

The SCFAs were then stratified into upper (≥75th percentile) and lower (≤25th percentile) quartiles, with the upper quartile referred to as high concentration and the lower quartile as low concentration, for evaluation of the association between SCFA levels and allergy-related outcomes. The ranges of 9 stool SCFAs concentration of the 25th and 75th percentiles are shown in the Supplementary Table 5.

Supplementary Figures 1, 2 depict the association of subjects with SCFAs in the 25th and 75th percentiles and the prevalence of allergy-related outcomes within each group. Generally, there are more positive cases within the 25th percentile group than within the 75th percentile group across all the timepoints.

### Association Between Stool SCFAs and Allergy-Related Outcomes

Children with low stool butyric acid levels (≤ 25 percentile) at 3 and 6 month time points had an increased odds ratio (OR) for eczema up to 8 years (adjOR (95% CI) = 58.3 (1.5–2315.7) and 41.9 (1.0–1,678.9) respectively) compared to children with higher stool butyric acid levels (≥75 percentile). A similar trend was observed between lower stool levels of acetic acid at 6 months with eczema up to 8 years [adjOR (95% CI) = 18.5 (1.0–336.2)] (*p* < 0.05).

Children with lower levels of caproic acid at 12 months were found to have higher odds of wheezing up to 8 years compared to children with higher levels [adjOR (95% CI) = 28.1 (1.2–650.3)]. Longitudinal analysis was carried out to further evaluate these associations.

When longitudinal analysis of the first 3 time points (3 weeks, 3 and 6 months) were taken into account, our findings showed that children with low butyric acid levels longitudinally were more likely to have wheezing (adjOR (95% CI) = 14.6 (1.2-189.7)), eczema [adjOR (95% CI) = 13.2 (1.1–158.3)], food sensitization [adjOR (95% CI) = 12.3 (1.3–115.0)] and combined outcomes of both wheezing and eczema [adjOR (95% CI) = 22.6 (1.3-382.7)] up to 8 years. These significant longitudinal results were lost when the 12-month time point was included in the analysis.

Low levels of propionic acid levels were associated with recurrent wheezing as seen in nine out of 62 up till age 8 years [adjOR (95% CI = 7.4 (1.2–44.2)] for all 4 time points (week 3, months 3, 6 and 12).

**Figure 3:**
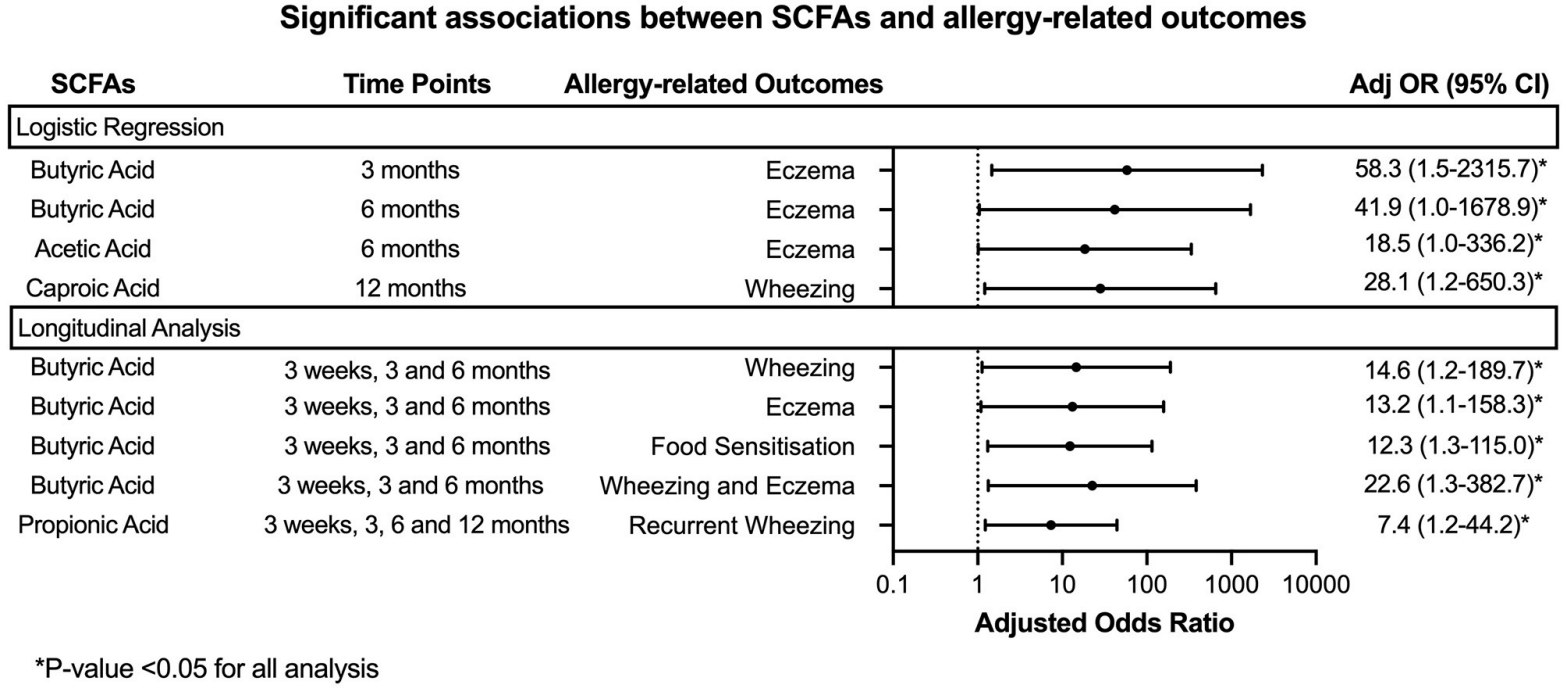
Forest plot denoting the significant results from the analysis of stool SCFAs with allergy-related outcomes. Logistic regression and Generalized Linear Mixed Models were employed to analyse the association between SCFA concentration at each time point or longitudinally with the allergy-related outcomes. The analysis was adjusted for gender, presence of siblings, mode of delivery, family history of atopic diseases and feeding pattern (exclusively breastfeeding, partially breastfeeding or exclusively formula in the first 6 months of life).

The longitudinal results are shown in Figure 3.

There were no other significant associations observed between the other SCFAs and the allergy-related outcomes.

## Discussion

This study investigated the longitudinal association between stool SCFA profiles across 4 time points in first year of life with the risk of developing allergy-related outcomes up to 8 years. Our results showed that subjects with lower longitudinal stool SCFA levels specifically butyric and propionic acids, especially during the weaning period, have a higher propensity of developing allergy-related outcomes. We also observed similar trends in association with eczema when single time points were analyzed, though statistical significance was only seen at the 3- and 6-month time points. Recently, few studies indicated that a compromised microbiome in the first 100 days of life prevents the establishment of butyrate producers at the time of weaning (13, 17). Those observations reinforce the notion that the infant gut microbiome follows a successive pattern of microbial colonization in the first year of life. First, establishment of breast milk sugars consumers in the first months of life, followed by the establishment of butyrate producers or fibers/polyphenols consumers at the time of weaning (13, 18).

Interestingly, lower butyric acid levels in the first 6 months and before 12 months were associated with eczema, and statistical significance was lost when the 12-month time point was included. This could be explained by the recommended introduction of solid food around 6 months which could influence the SCFA levels afterwards (19). Our selected cohort also showed a mean weaning age at 5.5 months (Supplementary Table 4). This suggests that the strong influence of stool SCFA levels on eczema risk occurs mainly early in infancy before the introduction of solid food.

Our findings add to the body of evidence that lower stool butyric acid levels before 6 months is associated with increased risk of developing wheezing, eczema, food sensitization and combined outcomes of both wheezing and eczema up to age 8 years, compared to those with higher levels. Other studies have also demonstrated the association between lower levels of stool butyric acid in infancy and increased likelihood of eczema by 18 months (10), food sensitization, food allergy, inhalant allergen and allergic rhinitis by 6 years (11). Additionally, we observed that higher odds of developing recurrent wheezing till age 8 years was associated with lower longitudinal levels of propionic acid, which has not been observed in previous studies. However, lower levels of stool propionic acid have been associated with inhalant allergen sensitization by 6 years (11).

Although the associations between lower levels of acetic acid with atopic wheeze at 12 months of age (12), with food sensitization and food allergy by 6 years (11) have been reported, we did not observe any significant association in terms of acetic acid levels with any of the allergy-related outcome. Taken together, these studies provide robust evidence for the protective effect of higher stool levels of SCFAs, particularly butyric acid between 3 and 6 months of age, against allergy-related outcomes. A preclinical study defined the “weaning reaction” as a sensitive period allowing the establishment of butyrate producers which have a paramount role in programming the immune system. Thus, early life provides a potential intervention window through a modulation of the infant gut microbiome (20). Table 1 summarizes the studies to date that have made similar observations to our study.

**Table 1:** Summary of the current cohort studies on the relation between stool SCFA levels and allergy-related outcomes.

**Cohort Name**	**SCFAs/bacteria**	**Findings**	**Time point for stool samples**
**SCFAs**			
Protection against Allergy-Study in Rural Environments (11)	Propionic and butyric acids	High levels of propionic and butyric acids were associated with less food sensitization and reduced likelihood of inhalant allergen by 6 years.	12 months
	Butyric acid	High levels of butyric acid showed a reduced likelihood of food allergy and allergic rhinitis by 6 years.	
	Acetic acid	High levels of acetic acid was associated with a reduced likelihood of food sensitization and food allergy by 6 years.	
Canadian Healthy Infant Longitudinal Development (CHILD) (12)	Acetic acid	Lower concentration of acetic acid was associated with atopic wheeze at 12 months of age.	3 months
PATCH cohort, with subjects from Australia, Singapore, England and Ireland (10)	Butyric acid	Lower levels of butyric acid was associated with eczema by 18 months.	26 weeks
Swedish BAS (BarnAllergiStudien or Pediatric Allergy Study) prospective birth cohort (8)	Valeric acid	The concentration of stool valeric acid at 12 months of age was inversely associated with eczema and food allergy at 13 years of age.	12 months
Swedish FARMFLORA birth cohort (9)	Valeric acid	Higher levels of stool valeric acids was positively associated with protection from eczema at 8 years.	3 years
	Isobutyric, isovaleric and valeric acids	High isobutyric, isovaleric and valeric acid levels were associated with lower rates of eczema, rhinitis, asthma and food allergy at 3 years.	
Swedish birth cohort study (7)	Isobutyric, isovaleric and valeric acids	Lower levels of stool isobutyric, isovaleric and valeric acids were associated with a higher likelihood of food allergy at 4 years.	12 months
Childhood Origin of Asthma and Allergic Diseases (COCOA) (6)	Butyric and valeric acids	Stool butyric and valeric acid were lower in infants with transient atopic dermatitis than in healthy controls and those with persistent atopic dermatitis. Transient AD was defined as a development of AD at 6 months of age and remission of this condition by 12 months of age, whereas persistent AD was defined as a development of AD at 6 months of age with persistent symptoms at 2 years of age.	6 months
Growing Up in Singapore Toward healthy Outcomes (GUSTO) (this study)	Butyric acid	Lower levels of butyric acid was positively associated with wheezing with/without concomitant eczema, eczema and food sensitization.	3 weeks, 3 and 6 months
	Propionic acid	Lower levels of stool propionic acid was associated with a higher likelihood of wheezing at 8 years.	3 weeks, 3, 6 and 12 months
**SCFA-producing bacteria**			
Europe PAPS cohort study (21)	*Lachnobacterium* and *Faecalibacterium*	A lower microbial diversity was associated with AD development at 3 years and allergic sensitization between 6 to 11 years, but not with asthma between 6 to 11 years. *Lachnobacterium* and *Faecalibacterium* were significantly decreased throughout infancy among children who developed AD at 3 years.	5, 13, 21 and 31 weeks
	*Lachnospira, Dialister* and *Lachnobacterium*	*Lachnospira* and *Dialister*, next to *Lachnobacterium*, were significantly decreased among children who developed asthma between 6 and 11 years.	
Subgroup of Estonian and Finiish children participating in the DIABIMMUNE (Pathogenesis of type 1 diabetes: testing the hygiene hypothesis) study (22)	Butyric acid producing *Faecalibacterium*	A decreased abundance of butyric acid producing *Faecalibacterium* at 3 months of age was associated with allergic diseases, particularly atopic sensitization, by age 3 years.	3 months
Copenhagen Prospective Studies on Asthma in Childhood (COPSAC) cohort (17)	Lachnospiraceae (including Lachnospiraceae incertae sedis and Roseburia) and Ruminococcaceae (including Ruminococcus and Faecalibacterium)	Children born to asthmatic mothers with lower relative abundances of these genera had an increased risk for asthma by age 5 years.	1 year

This notion is further substantiated by preclinical studies in mice, demonstrating that SCFAs such as acetic, butyric and propionic acids, produced in the gut can regulate homeostasis of Tregs in the colon (23, 24). These SCFAs have varying influence on Treg differentiation and accumulation due to varying degrees of interaction with different GPCRs that would then have different impact on immune cells (T cells and dendritic cells), hence conferring different immunological protection (24). These differential effects on the immune system may partly explain the different associations of each SCFA with different allergic outcomes. Evidence on protective effects and their mechanism of action for minor SCFAs is still lacking and thus deserves further study.

Current studies suggest that early life nutritional intervention could be a solution to modulate SCFA concentrations in the gut and reduce susceptibility to allergic diseases. Increased consumption of foods with SCFAs such as butyric acid in yogurt could be beneficial to directly increase gut SCFA concentration and hence increase levels of SCFAs in the body. This strategy has shown to be beneficial in the prevention of inflammation-related diseases such as chronic inflammatory bowel disease (25, 26). Evidence for the direct supplementation of SCFA intake in relation to the development of allergy-related outcomes remains sparse but deserves further attention.

Prebiotics and probiotics supplementation have been reported to promote the growth of SCFA-producers, which were shown to be associated with a beneficial effect against eczema (27–29). SCFA supplementation, particularly butyric acid, *via* oral supplements or consumption of food high in butyric acid, to treat colonic diseases has shown promising results in animal studies, with some of these effects being observed in human trials (25, 26). In relation to allergic disorders, preclinical studies in mice have also shown that fructo-oligosaccharide- (FOS-) supplemented diet and butyric acid supplementation improved efficacy of oral immunotherapy (OIT) for cow's milk allergy through the effective reduction of mast cell and basophil activation and enhanced suppressive activity of Tregs (30). These dietary interventions promote the growth of beneficial commensals, increase SCFA production, thus conferring immunological protective effects on the host (31). A translation of our findings into additional supportive clinical evidence could be alluded to epidemiological studies indicating that increased food diversity and higher intake of fruits and vegetables in the first years of life were associated with a robust immunity (less risk of allergy) (32–34).

The strength of this study is the serial assessment of stool SCFAs obtained in early life among children from a large birth cohort subjected to adjustment for various key confounding factors which have been known to affect the propensity of developing allergic outcomes. The long follow-up period of this cohort allowed for better monitoring of the trajectory of allergy manifestation as certain allergy-related disorders present later in childhood. The limitation of this study, however, is the relatively small sample size, and might be the reason for the few associations found with single time points of SCFAs, and wide confidence intervals with adjORs.

In conclusion, low levels of gut SCFAs, particularly butyric acid between 3 and 6 months of life and propionic acid up to 12 months are shown to be significantly associated with an increased susceptibility to allergic outcomes in childhood.

## Data Availability Statement

The datasets presented in this study can be found in online repositories. The names of the repository/repositories and accession number(s) can be found below: http://www.metabolomicsworkbench.org, PR000983.

## Ethics Statement

The studies involving human participants were reviewed and approved by Domain Specific Review Board of Singapore, National Healthcare Group and the Centralized Institutional Review Board of SingHealth (DSRB D/09/021 and CIRB 2009/280/D). Written informed consent to participate in this study was provided by the participants' legal guardian/next of kin.

## Author Contributions

HC performed the statistical analysis and manuscript writing. JC and DK carried out the experiment. GY carried out the data analysis. ET, EL, LS, NK, AG, HV, and OT were involved in the subject recruitment and sample collection. CH-H, YC, CL, and JK contributed to the interpretation of the results. FY, KT, Y-SC, and KG were involved in project administration. EC, BL, and LT were involved in planning and supervising the project. All authors contributed to the article and approved the submitted version.

## Funding

This work was supported by the Singapore Ministry of Health's National Medical Research Council [NMRC/CIRG/1414/2014].

## Conflict of Interest

CL and JK are employees of Danone Nutricia Research. The remaining authors declare that the research was conducted in the absence of any commercial or financial relationships that could be construed as a potential conflict of interest.

## Publisher's Note

All claims expressed in this article are solely those of the authors and do not necessarily represent those of their affiliated organizations, or those of the publisher, the editors and the reviewers. Any product that may be evaluated in this article, or claim that may be made by its manufacturer, is not guaranteed or endorsed by the publisher.
