# Supplementary material for: Evaluation of Stool Short Chain Fatty Acids Profiles in the First Year of Life With Childhood Atopy-Related Outcomes
Source: Front Allergy. 2022 Apr 6;3:873168. doi: 10.3389/falgy.2022.873168 (PMC9234937; doi:10.3389/falgy.2022.873168)
Supplement: Supplementary file 1 [file Data_Sheet_1.docx]

Supplementary Material

# Supplementary Data

Supplementary Table 1. Longitudinal association between major SCFAs (acetic, propionic and butyric acids) with allergy-related outcomes. * denotes significant results where p<0.05.

|  | | **Acetic acid** | | **Propionic acid** | | **Butyric acid** | |
| --- | --- | --- | --- | --- | --- | --- | --- |
|  |  | **Exp (B) (Lower bound - Upper bound)** | **Adj P-value** | **Exp (B) (Lower bound - Upper bound)** | **Adj P-value** | **Exp (B) (Lower bound - Upper bound)** | **Adj P-value** |
| **Cumulative wheezing up to 8 years** | **3 time points** | 2.8 (0.4-21.5) |  | 8.2 (1.0-68.6) |  | 14.6 (1.1-189.7) | * |
|  | **4 time points** | 1.7 (0.3-8.8) |  | 4.9 (0.8-29.1) |  | 2.4 (0.5-11.9) |  |
| **Cumulative eczema up to 8 years** | **3 time points** | 2.5 (0.4-15.6) |  | 2.6 (0.4-16.6) |  | 13.2 (1.1-158.3) | * |
|  | **4 time points** | 1.6 (0.3-7.2) |  | 1.6 (0.4-7.5) |  | 2.9 (0.6-14.1) |  |
| **Cumulative any SPT up to 8 years** | **3 time points** | 1.8 (0.3-11.1) |  | 2.0 (0.3-12.1) |  | 1.5 (0.2-12.4) |  |
|  | **4 time points** | 2.1 (0.5-9.0) |  | 1.4 (0.3-6.2) |  | 1.3 (0.3-5.9) |  |
| **Cumulative dust SPT up to 8 years** | **3 time points** | 1.8 (0.3-11.1) |  | 2.1 (0.3-12.1) |  | 1.5 (0.2-12.4) |  |
|  | **4 time points** | 2.1 (0.5-9.0) |  | 1.4 (0.3-6.2) |  | 1.3 (0.0-5.9) |  |
| **Cumulative food SPT up to 8 years** | **3 time points** | 2.5 (0.2-26.8) |  | 5.0 (0.2-111.0) |  | 12.3 (1.3-115.0) | * |
|  | **4 time points** | 3.9 (0.6-26.8) |  | 2.8 (0.4-18.9) |  | 5.9 (0.9-39.8) |  |
| **Cumulative eczema and wheezing up to 8 years** | **3 time points** | 3.8 (0.4-36.9) |  | 7.1 (0.7-70.9) |  | 22.6 (1.3-382.7) | * |
|  | **4 time points** | 1.7 (0.3-11.9) |  | 3.8 (0.5-28.8) |  | 3.1 (0.5-18.1) |  |
| **Cumulative recurrent wheezing up to 8 years** | **3 time points** | 1.6 (0.1-24.4) |  | 14.6 (0.3-614.9) |  | 1661490621.5 (0.0-.) |  |
|  | **4 time points** | 2.0 (0.2-21.2) |  | 7.4 (1.2-44.2) | * | 5.4 (0.2-138.9) |  |

Supplementary Table 2. Longitudinal association between minor SCFAs (isobutyric, valeric and isovaleric acids) with allergy-related outcomes.

|  | | **Isobutyric acid** | | **Valeric acid** | | **Isovaleric acid** | |
| --- | --- | --- | --- | --- | --- | --- | --- |
|  |  | **Exp (B) (Lower bound - Upper bound)** | **Adj P-value** | **Exp (B) (Lower bound - Upper bound)** | **Adj P-value** | **Exp (B) (Lower bound - Upper bound)** | **Adj P-value** |
| **Cumulative wheezing up to 8 years** | **3 time points** | 4.6 (0.5-38.2) |  | 7.3 (0.6-88.9) |  | 9.6 (0.7-137.5) |  |
|  | **4 time points** | 3.1 (0.6-17.6) |  | 3.2 (0.5-21.2) |  | 3.3 (0.6-18.1) |  |
| **Cumulative eczema up to 8 years** | **3 time points** | 4.1 (0.6-27.6) |  | 2.2 (0.3-16.0) |  | 4.1 (0.4-37.6) |  |
|  | **4 time points** | 3.6 (0.7-18.1) |  | 1.2 (0.2-6.8) |  | 2.9 (0.6-14.5) |  |
| **Cumulative any SPT up to 8 years** | **3 time points** | 0.9 (0.1-6.1) |  | 2.1 (0.3-16.9 |  | 0.5 (0.1-4.1) |  |
|  | **4 time points** | 1.2 (0.3-5.9) |  | 2.5 (0.4-16.2) |  | 0.7 (0.1-3.3) |  |
| **Cumulative dust SPT up to 8 years** | **3 time points** | 0.9 (0.1-6.1) |  | 2.1 (0.3-16.9) |  | 0.5 (0.1-4.1) |  |
|  | **4 time points** | 1.2 (0.3-5.9) |  | 2.5 (0.4-16.2) |  | 0.7 (0.1-3.3) |  |
| **Cumulative food SPT up to 8 years** | **3 time points** | 5.7 (0.5-63.9) |  | 21.4 (0.8-572.5) |  | 6061968559714260.0 (0.0-.) |  |
|  | **4 time points** | 5.5 (0.6-46.0) |  | 7.1 (0.9-57.5) |  | 6.8 (0.6-83.8) |  |
| **Cumulative eczema and wheezing up to 8 years** | **3 time points** | 8.9 (0.7-115.4) |  | 14.6 (0.6-369.8) |  | 14.8 (0.6-340.6) |  |
|  | **4 time points** | 5.2 (0.6-45.2) |  | 2.9 (0.3-27.1) |  | 4.3 (0.6-32.7) |  |
| **Cumulative recurrent wheezing up to 8 years** | **3 time points** | 4.0 (0.2-82.9) |  | 0.4 (0.0-25.5) |  | 3.1 (0.3-34.5) |  |
|  | **4 time points** | 5.9 (0.8-44.6) |  | 2.4 (0.2-32.1) |  | 5.6 (0.9-35.4) |  |

Supplementary Table 3. Longitudinal association between minor SCFAs (2-methylbutyric, caproic and 4-methylvaleric acids) with allergy-related outcomes.

|  | | **2-Methylbutyric acid** | | **Caproic acid** | | **4-Methylvaleric acid** | |
| --- | --- | --- | --- | --- | --- | --- | --- |
|  |  | **Exp (B) (Lower bound - Upper bound)** | **Adj P-value** | **Exp (B) (Lower bound - Upper bound)** | **Adj P-value** | **Exp (B) (Lower bound - Upper bound)** | **Adj P-value** |
| **Cumulative wheezing up to 8 years** | **3 time points** | 5.6 (0.4-83.8) |  | 1.7 (0.3-11.3) |  | 3.1 (0.5-18.0) |  |
|  | **4 time points** | 4.1 (0.6-28.7) |  | 2.4 (0.5-11.6) |  | 4.0 (0.8-20.3) |  |
| **Cumulative eczema up to 8 years** | **3 time points** | 3.8 (0.4-36.3) |  | 1.1 (0.2-6.5) |  | 1.9 (0.4-10.1) |  |
|  | **4 time points** | 2.5 (0.4-14.2) |  | 1.4 (0.3-6.3) |  | 2.6 (0.7-10.7) |  |
| **Cumulative any SPT up to 8 years** | **3 time points** | 0.6 (0.1-4.5) |  | 0.6 (0.1-3.7) |  | 1.1 (0.2-6.4) |  |
|  | **4 time points** | 0.8 (0.1-3.9) |  | 0.9 (0.2-4.1) |  | 1.2 (0.3-5.5) |  |
| **Cumulative dust SPT up to 8 years** | **3 time points** | 0.6 (0.1-4.5) |  | 0.6 (0.1-3.7) |  | 1.1 (0.2-6.4) |  |
|  | **4 time points** | 0.8 (0.1-3.9) |  | 0.9 (0.2-4.1) |  | 1.2 (0.3-5.5) |  |
| **Cumulative food SPT up to 8 years** | **3 time points** | 3.2 (0.3-30.2) |  | 2.0 (0.1-27.1) |  | 2.1 (0.3-17.6) |  |
|  | **4 time points** | 3.9 (0.5-32.4) |  | 2.5 (0.4-16.3) |  | 1.7 (0.3-11.0) |  |
| **Cumulative eczema and wheezing up to 8 years** | **3 time points** | 18.7 (0.4-869.9) |  | 1.9 (0.2-15.3) |  | 3.3 (0.4-28.2) |  |
|  | **4 time points** | 4.4 (0.4-46.7) |  | 2.4 (0.4-13.4) |  | 4.9 (0.8-30.3) |  |
| **Cumulative recurrent wheezing up to 8 years** | **3 time points** | 3.5 (0.2-72.8) |  | 1.2 (0.1-15.0) |  | 2.1 (0.3-13.2) |  |
|  | **4 time points** | 4.6 (0.6-35.2) |  | 3.1 (0.4-24.3) |  | 4.4 (0.5-43.3) |  |

Supplementary Table 4. Demographic and lifestyle characteristics of selected sub-cohort (n=75) and larger cohort (n=829).

|  | **Selected Sub-cohort^†^** | | | **Larger Cohort^†^** | | |
| --- | --- | --- | --- | --- | --- | --- |
|  | **N = 75** | | | **N = 829** | | |
|  | **n** | **%** |  | **n** | **%** |  |
| **DEMOGRAPHIC** |  |  |  |  |  |  |
| **Male gender** | 38 | (50.67) |  | 441 | (53.20) |  |
| **Presence of sibling** | 46 | (61.33) |  | 451 | (54.40) |  |
| **Caesarean delivery** | 25 | (33.33) |  | 249 | (30.07) |  |
| **Pre-term gestation (≥37 week)** | 72 | (96.00) |  | 775 | (93.49) |  |
| **Maternal antibiotics during labor** | 24 | (32.43) |  | 239 | (29.00) |  |
| **Maternal antibiotics during labor and/or pregnancy** | 36 | (49.32) |  | 348 | (44.67) |  |
| **Post-natal antibiotics within first year** | 17 | (22.97) |  | 337 | (47.40) | * |
| **Ethnicity** |  |  |  |  |  |  |
| Chinese | 42 | (56.00) |  | 482 | (58.14) |  |
| Malay | 21 | (28.00) |  | 205 | (24.73) |  |
| Indian | 12 | (16.00) |  | 142 | (17.13) |  |
| **Infant care attendance within first year** | 9 | (12.00) |  | 81 | (10.28) |  |
| **Feeding history in 1^st^ 6 months** |  |  |  |  |  |  |
| Exclusive breastfeeding | 3 | (4.00) |  | 84 | (11.40) | * |
| Partial breastfeeding | 63 | (84.00) |  | 473 | (64.18) |  |
| Exclusively formula | 9 | (12.00) |  | 180 | (24.42) |  |
| **Weaning Age – Mean ± SD (Range)** | 5.5 ± 1.0 (3.0-7.0) | | | 5.6 ± 1.2 (0.0-9.0) | | |
| **Family history of atopic diseases** | 26 | (46.43) |  | 295 | (50.86) |  |
| **Maternal history** |  |  |  |  |  |  |
| Any of allergic diseases | 18 | (24.66) |  | 178 | (23.89) |  |
| Asthma | 9 | (12.33) |  | 70 | (9.41) |  |
| Rhinitis | 8 | (10.96) |  | 96 | (12.89) |  |
| Eczema | 10 | (13.70) |  | 61 | (8.20) |  |
| **Paternal History** |  |  |  |  |  |  |
| Any of allergic diseases | 20 | (27.40) |  | 162 | (21.74) |  |
| Asthma | 10 | (13.70) |  | 60 | (8.06) |  |
| Rhinitis | 8 | (10.96) |  | 93 | (12.48) |  |
| Eczema | 7 | (9.60) |  | 39 | (5.20) |  |
| **Allergy-related Outcomes** |  |  |  |  |  |  |
| **Eczema** | 35 | (51.5) |  | 242 | (43.6) |  |
| **Non-Eczema** | 33 | (48.5) |  | 313 | (56.4) |  |
| **Wheezing** | 19 | (30.6) |  | 209 | (40.7) |  |
| **Non-Wheezing** | 43 | (69.4) |  | 305 | (59.3) |  |
| **Inhalant sensitisation** | 45 | (70.3) |  | 429 | (67.1) |  |
| **Non-Inhalant sensitised** | 19 | (29.7) |  | 210 | (32.9) |  |
| **Food sensitisation** | 17 | (27.0) |  | 95 | (18.1) |  |
| **Non-Food sensitised** | 46 | (73.0) |  | 430 | (81.9) |  |
| **Any sensitisation** | 45 | (69.2) |  | 441 | (68.5) |  |
| **No sensitisation** | 20 | (30.8) |  | 203 | (31.5) |  |

*Significant at *P*<0.05 compared with the larger remaining cohort group in chi square analysis.

*† Some variables had subjects with missing data.*

Supplementary Table 5. Minimum and maximum SCFA concentration (in nanomolar) in the 25^th^ and 75^th^ percentile range across week 3, months 3, 6 and 12.

| **SCFA** | **Week 3** | | | | **Month 3** | | | | **Month 6** | | | | **Month 12** | | | |
| --- | --- | --- | --- | --- | --- | --- | --- | --- | --- | --- | --- | --- | --- | --- | --- | --- |
|  | **25th Percentile** | | **75th Percentile** | | **25th Percentile** | | **75th Percentile** | | **25th Percentile** | | **75th Percentile** | | **25th Percentile** | | **75th Percentile** | |
|  | **min** | **max** | **min** | **max** | **min** | **max** | **min** | **max** | **min** | **max** | **min** | **max** | **min** | **max** | **min** | **max** |
| **Acetic Acid** | 16488 | 211878 | 375775 | 910906 | 1604 | 401851 | 584418 | 1166030 | 44101 | 396877 | 582331 | 1308447 | 63867 | 533288 | 697852 | 1365976 |
| **Propionic Acid** | 68 | 17733 | 34903 | 123707 | 1640 | 36760 | 658 | 228820 | 1974 | 37160 | 78121 | 164064 | 6940 | 57448 | 88530 | 233407 |
| **Butyric Acid** | 4 | 2149 | 16336 | 51494 | 41 | 12531 | 3.8693 | 107654 | 287.2 | 27701 | 73015 | 187378 | 437 | 52403 | 77080 | 447663 |
| **Isobutyric Acid** | 2 | 112 | 1934 | 25055 | 27 | 1992 | 17004 | 49372 | 11 | 1382 | 27775 | 109388 | 278 | 9436 | 28172 | 85371 |
| **Valeric Acid** | 0 | 92 | 480 | 11128 | 1 | 299 | 1430 | 30102 | 5.7 | 792 | 4549 | 87641 | 9 | 2141 | 6344 | 57645 |
| **Isovaleric Acid** | 2 | 55 | 834 | 12004 | 1 | 844 | 7985 | 33942 | 0 | 925 | 13484 | 64106 | 83 | 2991 | 15928 | 59950 |
| **2-methyl butyric Acid** | 0 | 12 | 389 | 6068 | 1 | 560 | 3936 | 16852 | 8 | 590 | 6441 | 28246 | 31 | 1846 | 7711 | 28287 |
| **Caproic Acid** | 1 | 17 | 104 | 1105 | 0 | 7 | 69 | 7698 | 0 | 5 | 191 | 11657 | 0 | 19 | 286 | 12553 |
| **4-methyl valeric Acid** | 0 | 0 | 200 | 17058 | 0 | 1.7 | 1132 | 21080 | 0 | 3.3 | 3819 | 57846 | 0 | 81 | 3597 | 29138 |


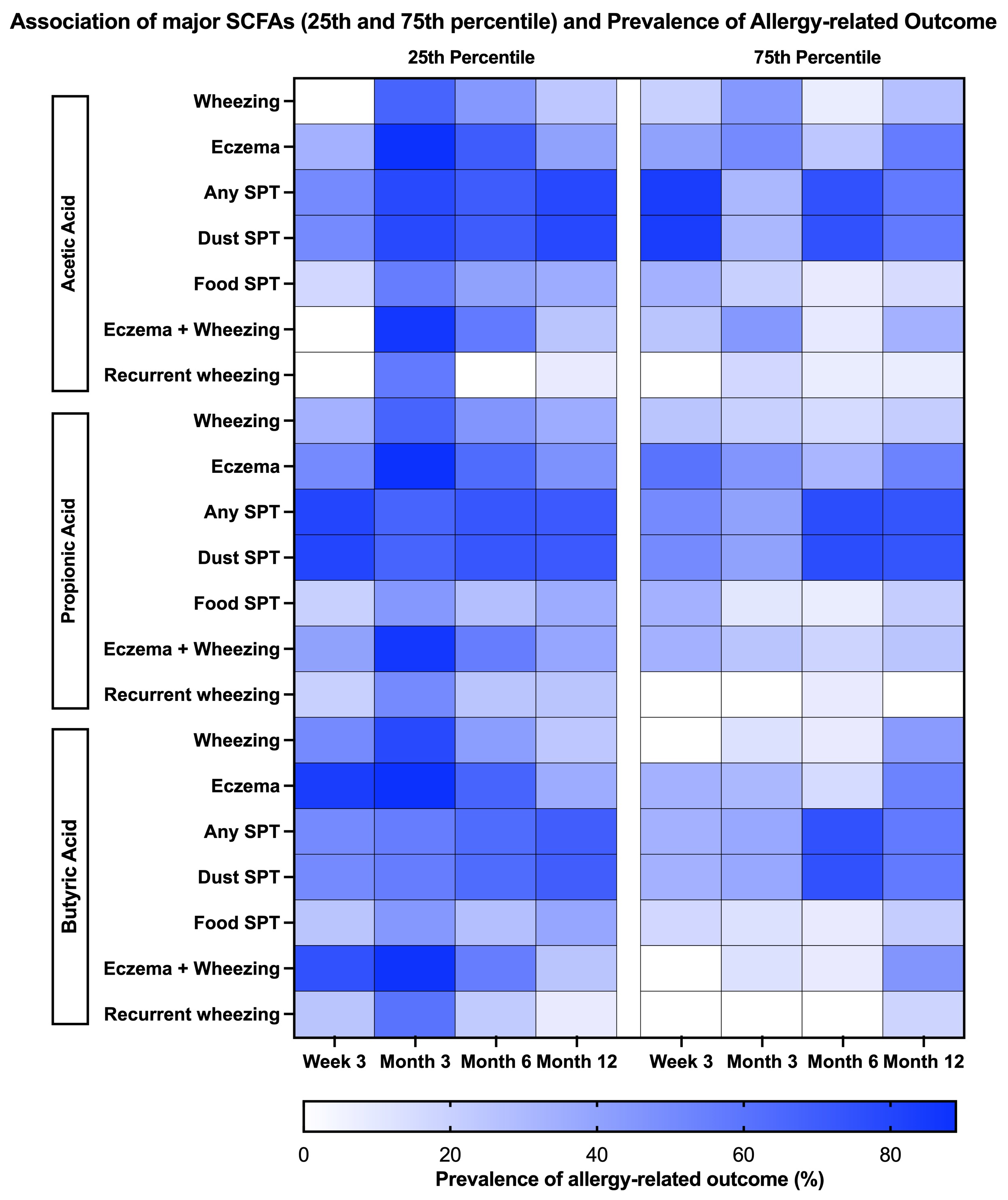


**Supplementary Figure 1. The association of subjects with major SCFAs (acetic, propionic and butyric acids) in the 25^th^ and 75^th^ percentiles and the prevalence of allergy-related outcomes. There is a lower prevalence of allergy-related outcome in the 75^th^ percentile group as compared to the 25^th^ percentile group at all timepoints for most SCFAs and outcomes.**


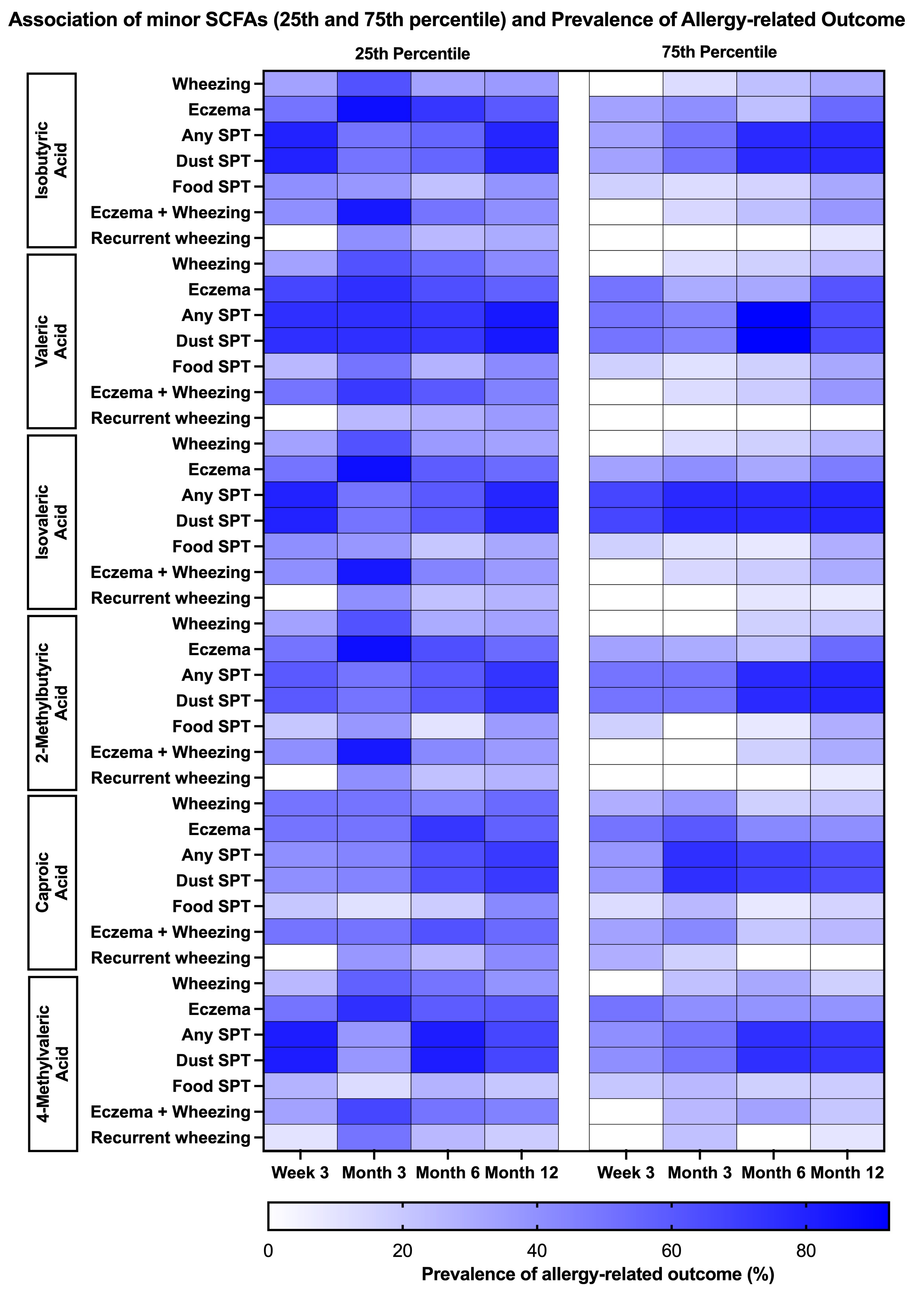


**Supplementary Figure 2. The association of subjects with major SCFAs (isobutyric, valeric, isovaleric, 2-methylbutyric, caproic, 4-methylvaleric acids) in the 25^th^ and 75^th^ percentiles and the prevalence of allergy-related outcomes. There is a lower prevalence of allergy-related outcome in the 75^th^ percentile group as compared to the 25^th^ percentile group at all timepoints for most SCFAs and outcomes.**
